# Restoring the unrestorable! Developing coronal tooth tissue with a minimally invasive surgical extrusion technique

**DOI:** 10.1038/s41415-019-0268-9

**Published:** 2019-05-24

**Authors:** Thomas Dietrich, Ralf Krug, Gabriel Krastl, Phillip L. Tomson

**Affiliations:** 1 The School of Dentistry, University of Birmingham, Birmingham, UK; Birmingham Community Healthcare NHS Foundation Trust, Birmingham, UK; 2 Department of Conservative Dentistry and Periodontology, University of Würzburg, Würzburg, Germany

## Abstract

Surgical extrusion is a recognised treatment option for teeth that have insufficient coronal tooth structure remaining due to deep caries, resorption or traumatic injury. However, the technique has not been widely adopted, arguably because extraction of a severely compromised tooth may be difficult to achieve in a gentle and predictable way. In this paper, we present our novel approach to surgical extrusion and subsequent management of teeth using a vertical extraction system (Benex), which has become the method of choice in the authors' practice for many teeth that would otherwise be deemed unrestorable. We describe the clinical procedure in detail and discuss the advantages and disadvantages compared to alternative approaches, including surgical crown lengthening and orthodontic extrusion.

## Key points

- Describes a technique for predictable surgical extrusion of teeth with deep subgingival lesions.
- Argues the technique offers an option for restoring these teeth, many of which would otherwise be deemed unrestorable.
- Discusses the advantages and limitations of the technique, in comparison with alternative approaches.

## Introduction

Teeth that have little coronal tooth structure or pathological features such as caries, resorption or a fracture line that extend deep into the gingival sulcus, are typically deemed non-restorable unless additional measures are taken to ensure that the restoration margins do not encroach on the biologic width. Available treatment options include surgical crown lengthening and orthodontic or surgical extrusion. While surgical crown lengthening is a predictable technique to ensure adequate biologic width, the procedure may result in unfavourable gingival architecture and compromised aesthetics. Orthodontic extrusion does not share these disadvantages, however it is relatively time consuming, costly and can result in unfavourable aesthetics for months. Surgical extrusion, or intra-alveolar transplantation, has been proposed as a third option to reposition the tooth into a more coronal position to allow restoration. The biological principles of surgical extrusion are well established and favourable outcomes have been reported.^1^,^2^,^3^,^4^ The technique has not been widely adopted, presumably due to the limited predictability of extracting a severely compromised tooth or root using conventional extraction techniques. Conventional extraction techniques using luxators, elevators or periotomes, rely on socket expansion and may result in significant trauma to the periodontal tissues including bone and/or further damage to the tooth root, such as fractures. Hence, the main challenges during tooth extraction in the context of surgical extrusion are to avoid further damage to the tooth/root structure itself as well as undue trauma to alveolar bone and periodontal ligament (PDL), which may result in subsequent root resorption.^5^

The Benex extraction system (Benex; Helmut Zepf Medizintechnik GmbH, Seitingen-Oberflacht, Germany) is designed to extract teeth without expanding the alveolar bone by using vertical forces exclusively. This minimises trauma to the root surface and alveolar bone. We have described the clinical procedure to extract non-molar teeth with the Benex system in detail and have demonstrated that this procedure is highly predictable.^6^,^7^,^8^

For several years now, the authors have used the Benex system for the surgical extrusion of teeth that have had little coronal tooth structure, deep subgingival caries or crown fractures, as an alternative to crown lengthening, orthodontic extrusion or, in most cases, extraction in order to allow these teeth to be restored predictably.^4^,^9^ The purpose of this paper is to provide a detailed description and critical discussion of the clinical procedure and associated restorative aspects, as it has evolved in the authors' hands.

## Clinical procedure for surgical extrusion using the Benex system

**Fig. 1:**
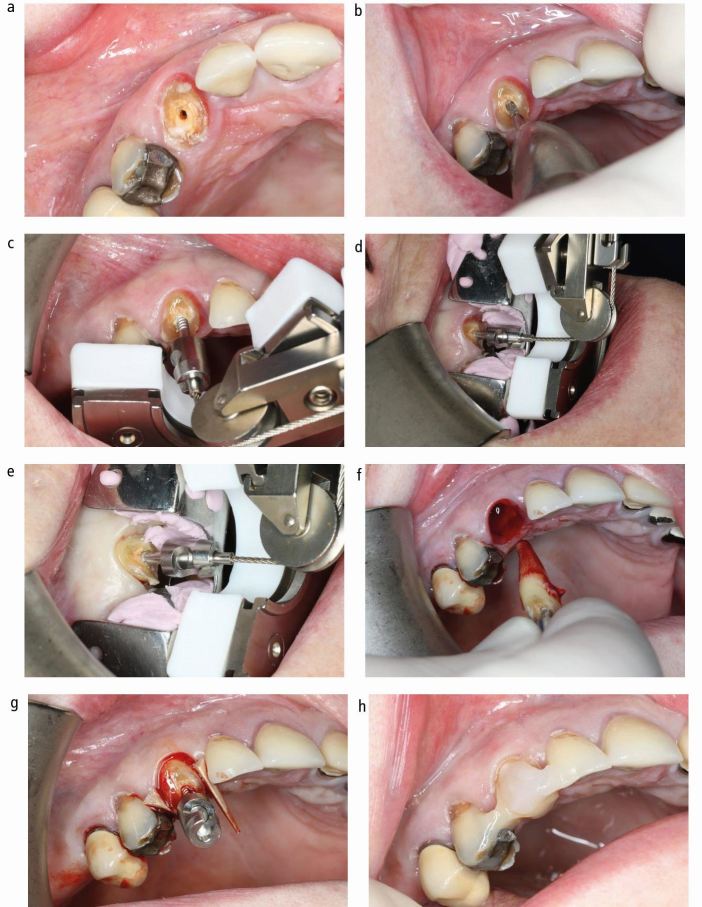
Surgical extrusion of upper right canine tooth. A) Deeply carious root with insufficient sound coronal tooth structure remaining for restoration; B) Preparation of Benex screw hole following caries removal; C) Application of the Benex extractor is possible, but the support tray should be used to ensure even distribution of extraction forces; D) Support tray and extractor in place, pullrope is perfectly aligned with long-axis of screw; E) Sharpey's fibre rupture has occurred and root is extruded; F) Root is removed from socket briefly to confirm absence of root fractures or perforation; G) Root repositioned in coronal position and secured with wooden wedges; H) extruded root splinted with composite

**Fig. 2:**
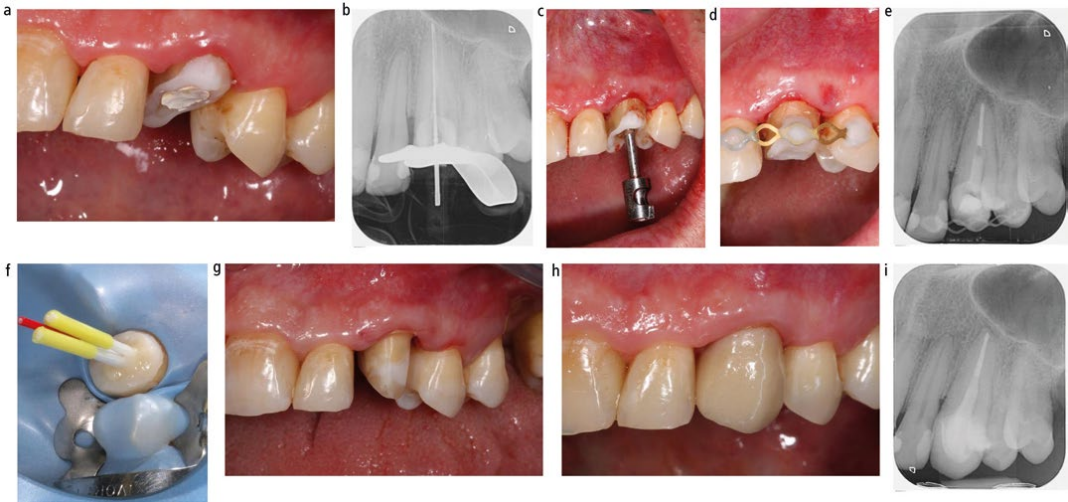
Surgical extrusion and restoration of upper left canine tooth. A) Root with temporary build-up to allow rubber dam placement for endodontic revision; B) Endodontic re-treatment; C) Surgical extrusion following successful endodontic revision; D) Root splinted in coronal position; E) Periapical radiograph showing apical radiolucency immediately after extrusion; F) Core-build up with fibre posts and composite; G) Preparation for full ceramic crown; H) Full ceramic crown cemented; I) Periapical radiograph 12 months postoperatively

The Benex system comprises a self-tapping screw that is anchored into the root canal. A vertical extraction force is then applied via a flexible pull rope using the extractor, resulting in severance of the dento-alveolar fibres and extraction of the root. Although the Benex system and its use for tooth extraction has been previously described in detail, there are a number of subtle but important differences to consider when using the system for extrusion rather than extraction, which will be discussed in the following step-by-step description of the procedure (Figs 1 and 2).^6^

### Caries removal

Removal of any remaining carious dentine is a critical first step in order to estimate the remaining volume of sound tooth tissue and allow for correct determination of the position of the final restoration margin. Depending on the type of final restoration planned, allowance should be made to create at least 2 mm of supragingival tissue for an appropriate ferrule design.

### Preparation of screw hole

The next step is the identification of the root canal and preparation of the screw hole. This process is akin to a post hole preparation when restoring a tooth with a post. Therefore, the usual precautions should be applied to avoid deviation from the root canal and minimise the risk of perforation. If the tooth already has a satisfactory root filling, we recommend removal of gutta-percha with a suitable drill before preparation of the screw hole with the matching diamond-coated Benex drill (Fig. 1b). The length of the diamond-coated area of the burs corresponds to the length of the screw thread. In contrast to an extraction, and depending on tooth type and any pre-existing root canal preparation, the depth of the preparation should be carefully considered and may not be to the full length of the bur/thread, in order to minimise hard tissue loss (Fig. 1c, d). For the same reason, the use of the thicker (2.1 mm) screw should be restricted to wide root canals where no retention can be obtained with the smaller diameter (1.6 mm) screw. The forces required to achieve root extraction vary widely from less than 100 N to more than 500 N. Full length preparation may be required in some cases to achieve luxation of the root.^7^,^10^

### Insertion of the screw

The matching self-tapping screw is inserted using the screwdriver to the appropriate length. Well cutting screws should be used for extrusion treatment to minimise the risk of root fracture, which may occur as the screw threads blunt. One easy way to keep track of screw usage is to keep two screws of each diameter/length in the Benex kit; one less used (no marking) and an older one (marked with a diamond bur on the screw head). The newer one is only used for extrusions and the older one for extractions. When the older screw gets blunt it is discarded and the other screw in the kit is now marked as old and replaced with a new screw.

### Placement of support tray

The use of the support tray is essential if the adjacent teeth are missing to allow application of the Benex extractor. Furthermore, use of the support tray helps to achieve axial alignment of the pull rope and therefore an ideal force vector, as well as distributing the extraction forces equally and thus avoiding complications due to excess forces on adjacent teeth. Following insertion of the Benex screw, the support tray is filled with a small amount of any heavy body silicone putty with low elasticity and put in place such that the tray is positioned perpendicularly to the long axis of the screw along the occlusal plane (Fig. 1d, e). The edges of the tray should not be in direct contact with the gingiva so that it does not traumatise the gingiva once pressure is applied.

### Placement of extractor and mounting the pull rope

The sledge of the extractor is brought into its base position and the ball end of the pull rope is engaged into the female component of the screw head. The wire rope is passed over the pulley and attached to the hook of the extraction sledge. While doing so, the pull rope should be kept under slight tension to ensure that the ball end does not detach from the screw head. The position of the extractor is now adjusted so that the screw (tooth axis) and pull rope are perfectly aligned (Fig. 1d, e).

### Extrusion

The extraction force is increased by slowly turning the handle clockwise. If and when significant resistance is felt by the operator, we advise to wait around 30 seconds before increasing the pulling force further. The forces required for extraction vary considerably, some roots will yield with minimal resistance felt by the operator when turning the handle, and others will require forces of over 500 N, which corresponds to the operator finding it difficult to turn the handle further. If the root does not yield after three to four minutes of pull time, a fine luxator can be used to luxate the root in a mesio-distal direction. In many cases, this can be achieved without dismounting the extractor. Unfortunately, in some cases of roots with significant curvature, undercuts or teeth with divergent roots (for example, some upper premolars), it will be impossible to extract/extrude (see discussion later in this article),^7^,^8^ and the extrusion procedure will have to be abandoned.

**Fig. 3:**
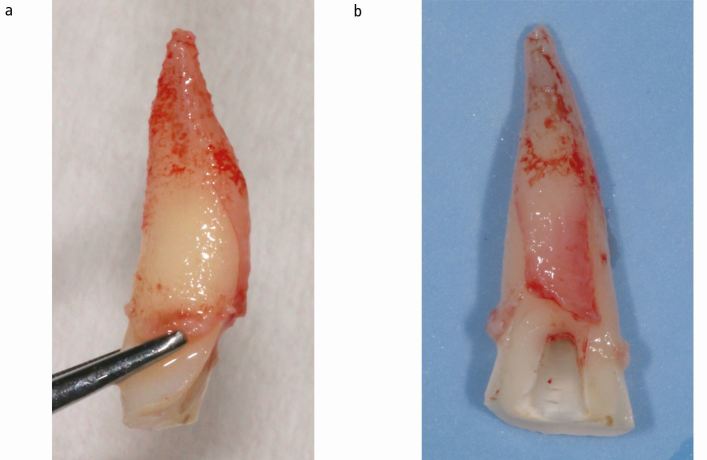
Upper central incisor with clinically diagnosed crown fracture. The tooth was considered for surgical extrusion. Following extraction with Benex and removal from the socket, a vertical root fracture was evident and the tooth deemed unrestorable

Rupture of dento-alveolar fibres and successful luxation will typically be indicated by mild bleeding from the sulcus and a drop in the tension in the pull rope (Fig. 1e). The handle screw is now slowly turned further until the root has reached the desired coronal position and the pull rope and extractor are dismounted. Alternatively, the root can now be removed from the socket by holding it by the screw head for a thorough visual inspection to confirm absence of perforations and/or root fractures (Fig. 1f). While this may not be necessary for some teeth, we would recommend it in cases of narrow diameter roots such as upper lateral or lower incisors, teeth that required high extraction forces, if there is the slightest doubt about the direction and depth of the screw canal preparation, and following traumatic injury (Fig. 3). Compared to conventional extraction techniques, the handling of the extracted/extruded root is technically much easier as the screw head serves as a convenient handle to manipulate the root as necessary, while minimising the risk of root surface contamination.

The root is first gently pushed back into the socket into its original position and the screw is loosened but not removed by turning it 90 degrees counter clockwise. The root is now positioned into its desired coronal position and stabilised with wooden wedges, followed by removal of the screw (Fig. 1g). The root is splinted to the neighbouring teeth. If root canal treatment has not been completed yet, the canal may be dressed and access secured with a temporary filling. If the root canal has already been obturated, core build-up with or without post placement can be performed, provided that satisfactory moisture control can be achieved. Splinting can be done before or after post and core construction. Depending on the situation and operator preference, this can be done with a titanium trauma splint, metal wire or with composite only (Fig. 1h and 2d).

Even after successful surgical extrusion, the amount of supragingival tooth substance is often limited. Thus, placement of a post may be necessary to provide adequate retention for the core and final restoration. Only minimal post space preparation should be carried out after BENEX extrusion, in order to avoid further tooth tissue removal and weakening of the tooth. Rather, a root canal post, which fills the post space created by the Benex preparation bur is luted with composite resin (Fig. 2f).

A mismatch between post and post space can be accepted and is not associated with reduced fracture resistance.^11^,^12^ Furthermore, the Benex canal thread grooves guarantee good post and core retention. As an alternative to conventional glass fibre posts, flexible and unpolymerised glass fibre posts (such as GC everStickPOST; GC Germany GmbH, Bad Homburg, Germany) can be individually adapted to the shape of the root canal. Special post drilling and cementing are no longer necessary.

Depending on the amount and the morphology of the defect, the final restoration can be accomplished either with a direct restoration or with a laboratory made crown. An adequate ferrule is essential to increase survival of the root canal-treated extruded tooth, if restored with a full crown (Fig. 2g).^13^

### Postoperative measures

The patient will receive the usual postoperative instructions. Oral hygiene in the area can be supplemented with chlorhexidine mouthwashes for one week, after which normal oral hygiene can resume. The patient is recalled three to six weeks following extrusion for splint removal and, if applicable, completion of endodontic treatment. Alternatively, endodontic treatment can be completed during the healing phase, provided that the location and type of splint allows adequate access and placement of rubber dam.

## Discussion

When faced with the challenge of restoring a tooth that has little coronal tissue, deep subgingival carious lesion or a root fracture, several alternative treatment options that allow tooth retention can be considered. Orthodontic extrusion is highly predictable and suitable for all tooth types; however, due to its cost, requirement for multiple appointments and unfavourable aesthetics it is not frequently used. Orthodontic extrusion will also lead to a coronal advancement of alveolar bone and gingiva, which may or may not be a desired effect. For molar teeth, surgical crown lengthening is typically the preferred option, as aesthetic considerations are less important and the anatomy of molar teeth is usually incompatible with surgical extrusion. For non-molar teeth, and in particular those in the aesthetic zone, surgical extrusion with the Benex system has become the preferred option in our clinics. However, its benefits and risks have to be carefully considered and weighted against those of surgical crown lengthening.

While surgical extrusion with the Benex device is a relatively quick and straightforward procedure which is completed in one session, there is a risk of failure. This may occur as a result of insufficient root length, untoward root morphology (such as hypercementosis), divergent roots (for example, upper premolars), slim roots (for example, lateral incisors) or technical mistakes that can result in the inability to extrude the root, root fractures or root perforations during the procedure.^7^ In a cohort of 323 non-molar teeth not suitable for forceps extraction, we have previously reported a failure risk of 14% (47/323 teeth) for Benex extractions.^8^ The risk of failure was higher in multi-rooted teeth and teeth with pre-existing root fillings. However, these data were obtained from a cohort of extraction cases,^8^ and the failure rate may be lower in teeth that are deemed restorable following extrusion.^4^ Upper premolars are a particular challenge, as it is typically impossible to gauge the likelihood of successful Benex extrusion preoperatively, unless a cone beam computer tomography is available that allows assessment of root morphology. In such cases, crown lengthening or orthodontic extrusion may be the preferred option.

Crown lengthening may also be more appropriate than extrusion in cases of interproximal caries with a largely intact clinical crown, which could then be more conservatively restored with, for example, a class II or class III restoration. Crown lengthening may also be more appropriate if a longer crown is actually desired for functional or aesthetic reasons.

In addition to immediate failures occurring during the extrusion procedure itself, root resorption is a recognised complication that may occur following surgical extrusion. This can range from non-progressive transient root resorption, which leads to an altered root contour but re-established PDL, to progressive root resorption, which includes infection-related root resorption and osseous replacement resorption (ankylosis). Data on the incidence of root resorption after surgical extrusion are scarce, and the existing literature is of only limited relevance to the technique described here. Firstly, it is important to distinguish between extrusions of teeth following traumatic injuries versus deep carious lesions. Clearly, in the case of traumatic injuries, it is impossible to know whether any resorption was the result of the primary insult to the PDL during the injury or the result of trauma associated with the extrusion. In a systematic review including 19 reports describing data of 243 teeth that were surgically extruded after traumatic crown fractures using conventional extraction techniques, the incidence of non-progressive and progressive root resorptions was 30% and 3%, respectively.^2^,^5^ Secondly, by obviating the need for lateral compression forces, the use of the Benex device minimises trauma to the PDL. We have previously reported an incidence of 10% of non-progressive root resorption and no progressive root resorption in a cohort of 51 teeth following Benex extrusions.^4^ The mean observation period in that study was 3.1 years, which is considered sufficient in order to detect even rare cases of late resorptions. However, mesio-distal luxation with an elevator was performed in all of these cases and may have contributed to some PDL trauma to induce such a response. We have found luxation to be rarely necessary,^7^ and as described above, do not recommend its routine use for extractions or extrusions.

For teeth that don't have a satisfactory root filling in place, a decision has to be made whether or not to perform the extrusion before or after root canal treatment. Performing the extrusion before root canal treatment has several important advantages. Firstly, the extrusion will obviate the frequent need for a temporary restoration to allow rubber dam placement for endodontic treatment and to obtain coronal seal. Secondly, as the extrusion itself may fail for a number of reasons, it should be performed first. This is particularly relevant for teeth with traumatic injuries, where inspection of the root outside the socket is recommended to rule out additional fractures (Fig. 3). Root canal treatment can be completed at any time, either during or after splinting. We only consider completion of root canal treatment before the extrusion if the feasibility of root canal treatment is questionable (for example, retreatments).

## Conclusion

We present here a clinical technique that we have used successfully for several years to retain and restore teeth that, in the vast majority of cases, would have otherwise been extracted. Although the principles of surgical extrusion have long been established, the availability of an extraction device that allows the predictable and minimally invasive extraction of roots has made surgical extrusion a genuine and frequently used treatment option for non-molar teeth with deep subgingival lesions due to caries or trauma.
